# Insights into the Understanding of the Nickel-Based Pre-Catalyst Effect on Urea Oxidation Reaction Activity

**DOI:** 10.3390/molecules29143321

**Published:** 2024-07-15

**Authors:** Haipeng Liu, Peike Wang, Xue Qi, Ao Yin, Yuxin Wang, Yang Ye, Jingjing Luo, Zhongqi Ren, Lina Chen, Suzhu Yu, Jun Wei

**Affiliations:** 1 Shenzhen Key Laboratory of Flexible Printed Electronics Technology, Harbin Institute of Technology (Shenzhen), Shenzhen 518055, China; ddxd_lhp@163.com (H.L.); 22s155067@stu.hit.edu.cn (P.W.); qixue@hit.edu.cn (X.Q.); yinao505@outlook.com (A.Y.); yxwang202104@126.com (Y.W.); 21s155114@stu.hit.edu.cn (Y.Y.); 21b355005@stu.hit.edu.cn (J.L.); zhong_qi_ren@163.com (Z.R.); 2 School of Materials Science and Engineering, Harbin Institute of Technology (Shenzhen), Shenzhen 518055, China; 3 State Key Laboratory of Advanced Welding and Joining, Harbin Institute of Technology (Shenzhen), Shenzhen 518055, China

**Keywords:** urea oxidation reaction, pre-catalyst, quantity of active sites, activity per active site

## Abstract

Nickel-based catalysts are regarded as the most excellent urea oxidation reaction (UOR) catalysts in alkaline media. Whatever kind of nickel-based catalysts is utilized to catalyze UOR, it is widely believed that the in situ-formed Ni^3+^ moieties are the true active sites and the as-utilized nickel-based catalysts just serve as pre-catalysts. Digging the pre-catalyst effect on the activity of Ni^3+^ moieties helps to better design nickel-based catalysts. Herein, five different anions of OH^−^, CO_3_^2−^, SiO_3_^2−^, MoO_4_^2−^, and WO_4_^2−^ were used to bond with Ni^2+^ to fabricate the pre-catalysts *β*-Ni(OH)_2_, Ni-CO_3_, Ni-SiO_3_, Ni-MoO_4_, and Ni-WO_4_. It is found that the true active sites of the five as-fabricated catalysts are the same in situ-formed Ni^3+^ moieties and the five as-fabricated catalysts demonstrate different UOR activity. Although the as-synthesized five catalysts just serve as the pre-catalysts, they determine the quantity of active sites and activity per active site, thus determining the catalytic activity of the catalysts. Among the five catalysts, the amorphous nickel tungstate exhibits the most superior activity per active site and can catalyze UOR to reach 158.10 mA·cm^–2^ at 1.6 V, exceeding the majority of catalysts. This work makes for a deeper understanding of the pre-catalyst effect on UOR activity and helps to better design nickel-based UOR catalysts.

## 1. Introduction

Substituting renewable energies for fossil fuels is an effective method to conquer energy and environment problems at the same time, which is an urgent need for our society [1,2,3,4,5,6]. However, renewable energies like solar and wind have drawbacks such as intermittency and transportation difficulties. Water electrolysis powered by renewable energies can transform these renewable energies into hydrogen molecules which can solve the drawbacks of renewable energies [7,8,9,10,11,12]. Water electrolysis is composed of the hydrogen evolution reaction (HER) at the anode and oxygen evolution reaction (OER) at the cathode [13,14,15]. Compared with HER, OER makes up the majority of energy waste in water electrolysis due to the inherent sluggish kinetics [16,17,18,19,20]. Changing the OER by the urea oxidation reaction (UOR) can extremely decrease the overpotential of overall water electrolysis, thus improving the utilization of renewable energies [1,3,10,19,20]. Therefore, the design of cheap and efficient UOR catalysts has received much attention from scientists.

Since the discovery of urea electrolysis, platinum and rhodium have been regarded as the most superior UOR catalysts [21]. The rare reserves and high cost of platinum and rhodium prompt the scientists to design new catalysts. Nickel-based catalysts bio-inspired from urease have received much attention since their discovery and they exhibit comparable UOR performance to precious metals platinum and rhodium [7,22,23,24,25]. In order to obtain more active nickel-based catalysts, various types of nickel-based catalysts were developed to catalyze UOR mainly spanning from nickel oxides [17,24,26,27,28,29,30], nickel X-ides (X refers to C, N, P, S, Se, Te, etc.) [3,11,22,25,31], nickel-based composites [9,23,32], etc. For nickel oxide-related catalysts, Chen’s group added Rh nanoparticles to NiO nanosheets which outperformed the counter NiO nanosheets [27]. Rh and NiO are both UOR active components, and the addition of Rh nanoparticles endows the NiO nanosheet with a superior electronic structure. For nickel X-ide-related catalysts, Zhang’s group supported nickel nitride nanospheres on nickel foam, which can efficiently catalyze the hydrogen generation and urea decomposition [31]. The superior electron conductivity of the support nickel foam brought out the catalytic performance of nickel nitride. For nickel-based composite-related catalysts, Hameed’s group developed a type of NiO/graphene composite by a facile coprecipitation and calcination method [33]. The introduction of graphene improves the conductivity of NiO and alters the electronic structure of NiO, and the NiO/graphene composite demonstrates more enhanced UOR activity than NiO. Through lots of in situ experimental research by advanced instruments and many theoretical investigations, although various types of nickel-based UOR catalysts are synthesized and demonstrate different UOR performances, it is widely believed that the as-synthesized catalysts just serve as the pre-catalysts and the in situ-formed Ni^3+^ moieties are the true active sites [24,25,34]. Since the true active sites are all Ni^3+^ moieties, we question why different nickel-based catalysts demonstrate different UOR activity and what the significance of synthesizing different types of nickel-based catalysts is Systematic investigation about the effect of pre-catalysts on UOR activity helps to understand the meaning of synthesizing different kinds of catalysts and will lay a great foundation for further enhancing the UOR performance of nickel-based catalysts.

In this work, five catalysts were synthesized by bonding Ni^2+^ with OH^−^, CO_3_^2−^, SiO_3_^2−^, MoO_4_^2−^ and WO_4_^2−^ anions, respectively. Modern characterization techniques were adopted to analyze the catalyst structure and lots of electrochemical methods were utilized to uncover the UOR mechanism. It is found that the as-fabricated five catalysts just serve as the pre-catalysts and the true active sites are the in situ-formed Ni^3+^ moieties. The pre-catalysts determine the UOR catalytic activity by deciding the quantity of active sites and activity per active site. The as-prepared amorphous nickel tungstate exhibits the most superior activity per active site and demonstrates superior UOR activity, selectivity, and durability. This work helps to better understand the UOR mechanism.

## 2. Results and Discussion

### 2.1. Structure Characterization

**Scheme 1:**
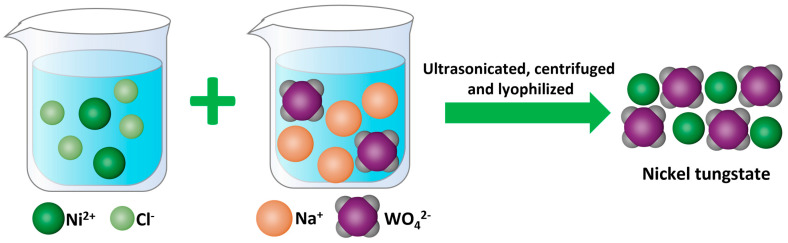
Synthetic scheme of Ni-WO_4_.

*β*-Ni(OH)_2_, Ni-CO_3_, Ni-SiO_3_, Ni-MoO_4_, and Ni-WO_4_ catalysts were fabricated according to Scheme 1, which is detailed in the Materials and Methods section. *β*-Ni(OH)_2_, Ni-CO_3_, Ni-SiO_3_, Ni-MoO_4_, and Ni-WO_4_ were obtained by mixing the Ni^2+^ solution with OH^−^, CO_3_^2−^, SiO_3_^2−^, MoO_4_^2−^, and WO_4_^2−^ solutions, respectively, followed by ultrasonication, centrifugation, and lyophilization.

**Figure 1:**
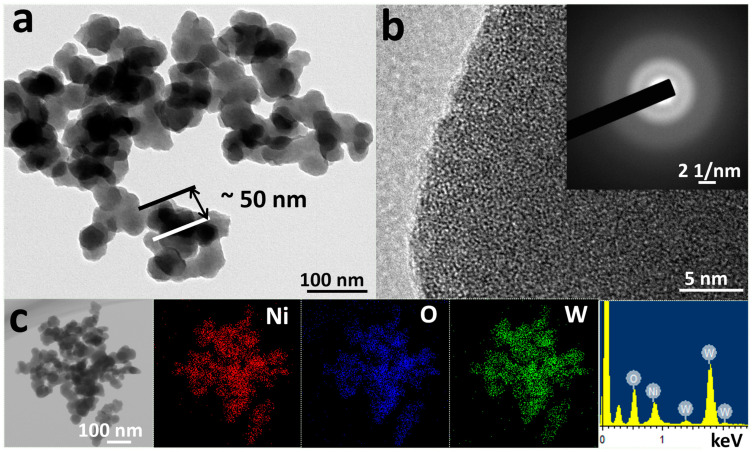
(**a**) TEM image, (**b**) HRTEM image, inset is SAED pattern, and (**c**) EDS mapping images and EDS diagram of Ni-WO_4_.

XRD was adopted to analyze the catalyst structure shown in Figure S1. *β*-Ni(OH)_2_ was poorly crystallized and typical peaks of (001), (100), and (011) facets located at 19.2, 33.2, and 38.6° were indexed to PDF#73–1520 [30]. XRD patterns of Ni-CO_3_, Ni-SiO_3_, Ni-MoO_4_, and Ni-WO_4_ all demonstrated amorphous hump peaks and the amorphous features were verified by their corresponding HRTEM images. TEM was used to observe the catalyst morphologies and analyze the detailed catalyst phase structure. *β*-Ni(OH)_2_, Ni-CO_3_, Ni-SiO_3_, Ni-MoO_4_, and Ni-WO_4_ all display nanoparticle morphologies (Figure 1a and Figure S2a–d). A (011) facet of 0.21 nm is observed in the HRTEM image of *β*-Ni(OH)_2_ and the selected area electron diffraction (SAED) pattern demonstrates typical polycrystalline diffraction ring feature (Figure S2e) [32,35,36]. No lattice planes can be detected in the HRTEM images of Ni-CO_3_, Ni-SiO_3_, Ni-MoO_4_, and Ni-WO_4_, and their corresponding SAED patterns all demonstrate amorphous halo features (Figure S2f–h and Figure 1b). Uniform distribution of elements Ni, O, and W can be seen in the EDS mapping images of Ni-WO_4_ and signals of Ni, O, and W are clearly detected in the EDS diagram of Ni-WO_4_ (Figure 1c). XRD and TEM results illustrate that *β*-Ni(OH)_2_ is a poorly crystallized nanoparticle, and Ni-CO_3_, Ni-SiO_3_, Ni-MoO_4_, and Ni-WO_4_ are amorphous nanoparticles.

**Figure 2:**
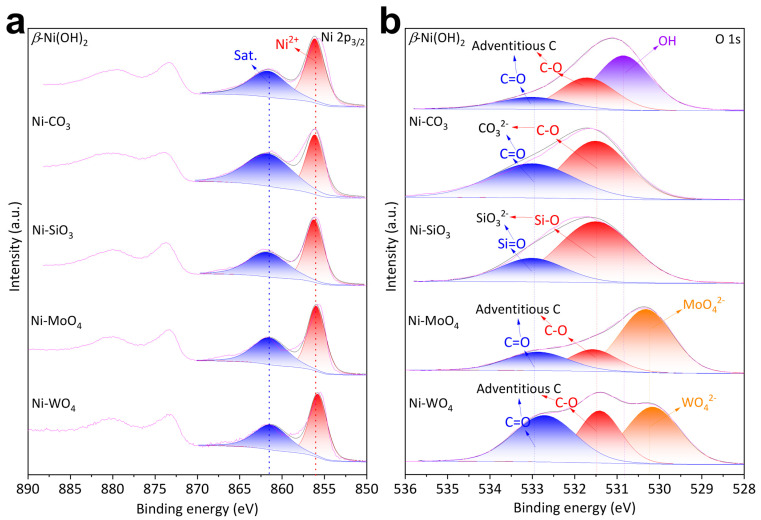
(**a**) Ni 2p XPS and (**b**) O 1s XPS of *β*-Ni(OH)_2_, Ni-CO_3_, Ni-SiO_3_, Ni-MoO_4_, and Ni-WO_4_.

Element chemical environments were characterized by XPS. Ni 2p_3/2_ XPS of *β*-Ni(OH)_2_, Ni-CO_3_, Ni-SiO_3_, Ni-MoO_4_, and Ni-WO_4_ were fitted by the moiety of Ni^2+^ (856.10 eV), followed by a satellite (861.60 eV) at a higher binding energy (Figure 2a) [12,36,37]. The fittings of O 1s XPS are different (Figure 2b). C-O moieties (531.70 eV) and C=O moieties (533.00 eV) from adventitious carbon and OH^−^ moieties (530.85 eV) from *β*-Ni(OH)_2_ structure were detected on *β*-Ni(OH)_2_ [30,36]. C-O moieties (531.50 eV) and C=O moieties (533.00 eV) from nickel carbonate structure were detected on Ni-CO_3_ [36,38,39]. Si-O moieties (531.50 eV) and Si=O moieties (533.00 eV) from nickel metasilicate structure were detected on Ni-SiO_3_ [40,41]. C-O moieties (531.70 eV) and C=O moieties (533.00 eV) from adventitious carbon and Mo-O moieties (530.31 eV) from nickel molybdate structures were detected on Ni-MoO_4_ [42,43]. C-O moieties (531.70 eV) and C=O moieties (533.00 eV) from adventitious carbon and W-O moieties (530.31 eV) from nickel tungstate structures were detected on Ni-WO_4_ [7,43]. Si 2p_3/2_ (102.45 eV) and 2p_1/2_ (103.06 eV) were detected on Ni-SiO_3_ (Figure S3a) [41]. Mo 3d_5/2_ (232.13 eV) and 3d_3/2_ (235.26 eV) were detected on Ni-MoO_4_ (Figure S3b) [42,44].

### 2.2. Electrochemical Analysis

**Figure 3:**
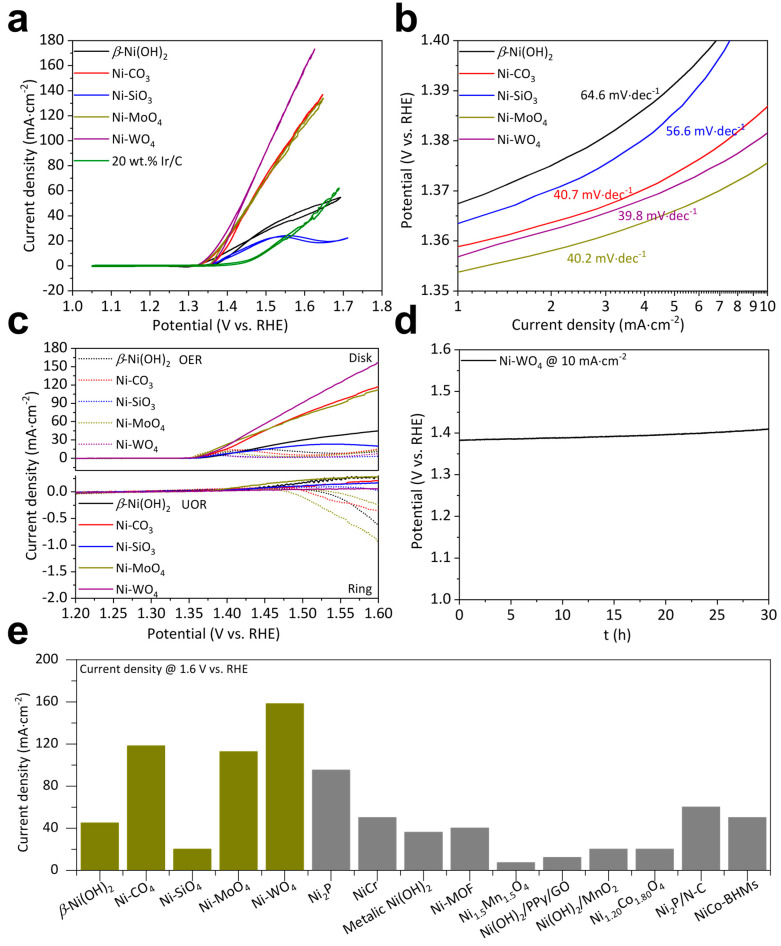
(**a**) UOR CV curves, (**b**) UOR Tafel slopes, and (**c**) OER and UOR RRDE polarization curves of *β*-Ni(OH)_2_, Ni-CO_3_, Ni-SiO_3_, Ni-MoO_4_, and Ni-WO_4_. (**d**) UOR durability of Ni-WO_4_. (**e**) Comparison of UOR performance of catalysts decorated on glassy carbon electrode.

Electrochemical measurements were conducted on electrochemical workstations. UOR CV curves of *β*-Ni(OH)_2_, Ni-CO_3_, Ni-SiO_3_, Ni-MoO_4_, and Ni-WO_4_ all demonstrate a more negative overpotential than those of OER at a fixed current density, while 20 wt.% Ir/C does not (Figure S4a). Therefore, the as-fabricated five catalysts are all applicable to UOR catalysis. *β*-Ni(OH)_2_, Ni-CO_3_, Ni-SiO_3_, Ni-MoO_4_, and Ni-WO_4_ start to catalyze UOR at a potential of 1.35, 1.34, 1.35, 1.35, and 1.36 V, respectively. *β*-Ni(OH)_2_, Ni-CO_3_, Ni-SiO_3_, Ni-MoO_4_, and Ni-WO_4_ deliver 10 mA·cm^–2^ at 1.41, 1.39, 1.42, 1.38, and 1.38 V, respectively. *β*-Ni(OH)_2_, Ni-CO_3_, Ni-SiO_3_, Ni-MoO_4_, and Ni-WO_4_ catalyze UOR at 1.6 V reaching 44.87, 117.69, 19.86, 112.60, and 158.10 mA·cm^–2^, respectively. Onset potentials and overpotentials at 10 mA·cm^–2^ do not show significant differences. But Ni-WO_4_ can catalyze UOR to reach an extremely higher current density at 1.6 V than *β*-Ni(OH)_2_, Ni-CO_3_, Ni-SiO_3_, and Ni-MoO_4_. UOR kinetics were examined by Tafel slope values. Ni-WO_4_ exhibits a lower value of 39.8 mV·dec^–1^ than *β*-Ni(OH)_2_ (64.6 mV·dec^–1^), Ni-CO_3_ (40.7 mV·dec^–1^), Ni-SiO_3_ (56.6 mV·dec^–1^), and Ni-MoO_4_ (40.2 mV·dec^–1^) (Figure 3b). UOR selectivity was performed on an RRDE equipment. The ring electrode demonstrates negligible current of the UOR RRDE curves, while the ring electrode exhibits obvious current of the OER RRDE curves, suggesting that O_2_ was produced during the OER test and no O_2_ was produced during the UOR test. Therefore, we can conclude that *β*-Ni(OH)_2_, Ni-CO_3_, Ni-SiO_3_, Ni-MoO_4_, and Ni-WO_4_ can effectively oxidize urea molecules in the tested solution. The UOR stability of Ni-WO_4_ was examined by the CP method. Ni-WO_4_ can catalyze the UOR for at least 30 h at 10 mA·cm^–2^ (Figure 3d). Catalyst support can effectively impact the catalyst activity. Therefore, UOR activity of the as-fabricated five catalysts is compared to catalysts decorated on the same support in previous publications. Impressively, the UOR activity of Ni-WO_4_ exceeds the majority of the superior catalysts (Figure 3e) [17,18,24,25,26,32,45,46,47,48].

### 2.3. Determining Factors of UOR Activity

**Figure 4:**
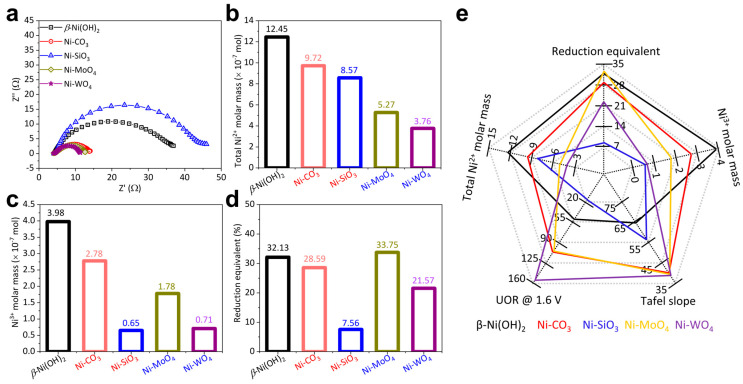
(**a**) UOR EIS, (**b**) total Ni^2+^ molar mass, (**c**) Ni^3+^ molar mass transformed from Ni^2+^, (**d**) reduction equivalent, and (**e**) summary of UOR performance of β-Ni(OH)_2_, Ni-CO_3_, Ni-SiO_3_, Ni-MoO_4_, and Ni-WO_4_.

Catalytic activity is the sum of activity per active site. Therefore, the activity per active site and the quantity of active sites co-determine the catalyst activity [49]. Charge transfer resistance reflecting the UOR barrier was evaluated by EIS [38]. The UOR EIS diameter follows this sequence: Ni-SiO_3_ < *β*-Ni(OH)_2_ < Ni-MoO_4_ < Ni-CO_3_ < Ni-WO_4_ (Figure 4a), suggesting that the UOR process proceeds more efficiently on Ni-WO_4_ than Ni-SiO_3_, Ni-MoO_4_, and Ni-CO_3_.

The quantity of active sites: It is widely believed that Ni^3+^ moieties are the true active sites and the pre-catalysts just serve as the precursors during UOR catalysis [11,25,31,50]. It is crucial to untangle the relationship among the Ni^2+^ molar mass, the Ni^3+^ molar mass, the reduction equivalent (the capacity to accumulate Ni^3+^) and UOR activity. The total Ni^2+^ molar mass (calculated from the catalyst loading on glass carbon electrode) follows this sequence: *β*-Ni(OH)_2_ > Ni-CO_3_ > Ni-SiO_3_ > Ni-MoO_4_ > Ni-WO_4_ (Figure 4b). The Ni^3+^ molar mass (calculated from the reduction peak in Figure S4b) follows this sequence: *β*-Ni(OH)_2_ > Ni-CO_3_ > Ni-MoO_4_ > Ni-WO_4_ > Ni-SiO_3_ (Figure 4c). The reduction equivalent follows this sequence: Ni-MoO_4_ > *β*-Ni(OH)_2_ > Ni-CO_3_ > Ni-WO_4_ > Ni-SiO_3_ (Figure 4d). The summary of the relationship among the Ni^2+^ molar mass, the Ni^3+^ molar mass, reduction equivalent and UOR activity is shown in Figure 4e. The Ni^2+^ molar mass, Ni^3+^ molar mass, reduction equivalent and UOR activity do not exhibit a reasonable logic relationship, indicating that the quantity of active sites cannot solely decide the UOR activity.

**Figure 5:**
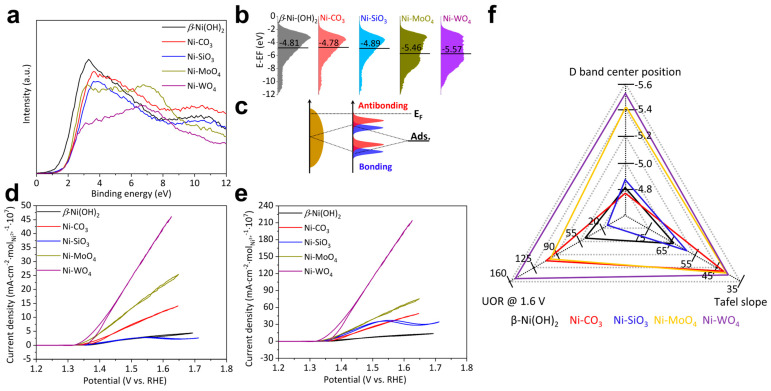
(**a**) Valence band spectra, (**b**) d band models derived from valence band spectra, (**c**) bond formation scheme, (**d**) UOR CV curves normalized by the total Ni^2+^ molar mass, (**e**) UOR CV curves normalized by Ni^3+^ molar mass, and (**f**) summary of UOR performance of β-Ni(OH)_2_, Ni-CO_3_, Ni-SiO_3_, Ni-MoO_4_, and Ni-WO_4_.

The activity per active site: The activity per active site is determined by the electronic structure of catalysts. The electronic structure was analyzed by valence band (VB) spectra shown in Figure 5a. VB spectra of *β*-Ni(OH)_2_, Ni-CO_3_, and Ni-SiO_3_ demonstrate similar shapes and are just located at slightly different binding energies. VB spectra of Ni-MoO_4_ and Ni-WO_4_ exhibit similar shapes but significantly differ from those of *β*-Ni(OH)_2_, Ni-CO_3_, and Ni-SiO_3_ especially below 6 eV. This indicates that Ni-MoO_4_ and Ni-WO_4_ possess significantly different electronic structures from *β*-Ni(OH)_2_, Ni-CO_3_, and Ni-SiO_3_ [7]. This phenomenon is due to the fact that transition metals mainly contribute below 6 eV in VB spectra [51]. D band centers were calculated from VB spectra (Figure 5b). D band center positions follow this sequence: Ni-CO_3_ (−4.78 eV) > *β*-Ni(OH)_2_ (−4.81 eV) > Ni-SiO_3_ (−4.89 eV) > Ni-MoO_4_ (−5.46 eV) > Ni-WO_4_ (−5.57 eV). The lower the d band center position, the weaker the bonding strength between the catalyst and intermediates. The bonding strength between the catalyst and intermediates subject to d band center position [51,52] and the bonding scheme are depicted in Figure 5c. The desorption of the UOR intermediates is the rate-controlling step and Ni-WO_4_ demonstrates the lowest d band center, which facilitates the desorption of the UOR intermediates. Therefore, Ni-WO_4_ exhibits the highest activity per active site. For the direct analysis of the activity per active site, the UOR CV curves were normalized by the Ni^2+^ and Ni^3+^ molar mass. The UOR activity per Ni^2+^ molar mass follows this sequence: *β*-Ni(OH)_2_ ≈ Ni-CO_3_ < Ni-SiO_3_ < Ni-MoO_4_ < Ni-WO_4_ (Figure 5d). The UOR activity per Ni^3+^ molar mass follows this sequence: Ni-CO_3_ < Ni-SiO_3_ < *β*-Ni(OH)_2_ < Ni-MoO_4_ < Ni-WO_4_ (Figure 5e). The summary of the total Ni^2+^ molar mass, Ni^3+^ molar mass, and UOR activity does not exhibit a reasonable logic relationship, suggesting that the activity per active site cannot solely determine the UOR activity as well (Figure 5f).

The activity per active site and the quantity of active sites co-determines the UOR activity of *β*-Ni(OH)_2_, Ni-CO_3_, Ni-SiO_3_, Ni-MoO_4_, and Ni-WO_4_. Ni-WO_4_ with the lowest d band center position demonstrates the highest activity per active site and possesses slightly fewer active sites compared to Ni-CO_3_ with the maximum quantity of active sites. Therefore, Ni-WO_4_ exhibits the highest UOR activity.

### 2.4. Effect of the Pre-Catalyst Catalyst on UOR Activity

**Figure 6:**
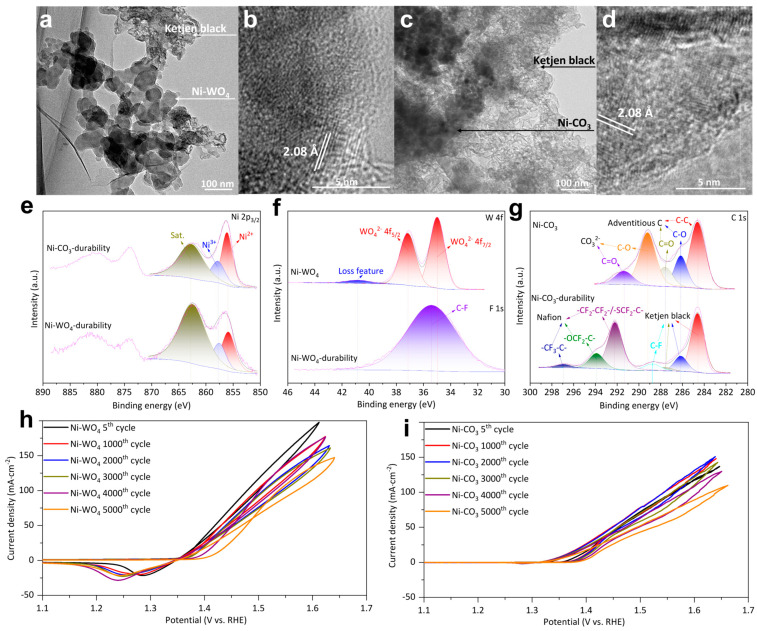
(**a**) TEM image and (**b**) HRTEM image of Ni-WO_4_ durability. (**c**) TEM image and (**d**) HRTEM image of Ni-CO_3_ durability. (**e**) Ni 2p XPS of Ni-CO_3_ durability and Ni-WO_4_ durability. (**f**) W 4f XPS of Ni-WO_4_ and F 1s XPS of Ni-WO_4_ durability. (**g**) C 1s XPS of Ni-CO_3_ and Ni-CO_3_ durability. (**h**) UOR CV curves of Ni-WO_4_. (**i**) UOR CV curves of Ni-CO_3_.

To verify if the true active sites are Ni^3+^ moieties for Ni-WO_4_ and Ni-CO_3_, TEM and XPS were adopted to examine Ni-WO_4_ durability and Ni-CO_3_ durability. Catalyst nanoparticles and Ketjen black are observed in TEM images of Ni-WO_4_ durability and Ni-CO_3_ durability (Figure 6a,c). HRTEM images of Ni-WO_4_ durability and Ni-CO_3_ durability both demonstrate a facet with a width of 2.08 Å ascribed to the (200) facet of nickel (oxy)hydroxide (Figure 6b,d) [22,35]. Ni 2p_3/2_ XPS of Ni-WO_4_ durability and Ni-CO_3_ durability are fitted with Ni^2+^ (856.10 eV) and Ni^3+^ (857.80 eV), followed by a satellite (862.70 eV) [36], confirming the formation of Ni^3+^ moieties. W 4f XPS of Ni-WO_4_ possesses three peaks of 4f_7/2_ (34.98 eV), 4f_5/2_ (37.16 eV), and tungsten oxide loss feature (40.81 eV) (Figure 6f) [53,54]. W signal cannot be detected in the corresponding binding energy range for Ni-WO_4_ durability, suggesting that element W on the Ni-WO_4_ surface was completely dissolved during UOR catalysis. F 1s XPS of Ni-WO_4_ durability demonstrates one peak of C-F moieties located at 35.39 eV which results from the Nafion molecules (Figure 6f) [55]. C-C (284.60 eV), C-O (286.14 eV), C=O (287.60 eV), C-O (289.29), and C=O (291.46) are detected in C 1s XPS of Ni-CO_3_ (Figure 6g) [55,56]. C-C (284.60 eV), C-O (286.14 eV), C=O (287.60 eV), C-F (288.77 eV), -CF_2_-CF_2_-/-SCF_2_-C- (292.19 eV), -OCF_2_-C- (293.88 eV), and -CF_3_-C- (296.89 eV) are detected in C 1s XPS of Ni-CO_3_ durability (Figure 6g) [55]. The comparison of C 1s and W 4f fitting results of Ni-CO_3_ and Ni-WO_4_ before and after UOR durability tests (Table S1). The signal of nickel carbonate structure in C 1s XPS spectrum of Ni-CO_3_ durability disappeared, indicating the complete dissolution of nickel carbonate on Ni-CO_3_ surface during UOR catalysis. The UOR activity of both Ni-WO_4_ and Ni-CO_3_ just slightly fluctuates during the continuous 5000 cycles (Figure 6h,i). Therefore, we can infer that the WO_4_^2−^ and CO_3_^2−^ do not directly participate in the UOR catalysis and true active sites are Ni^3+^ moieties. The higher UOR activity of Ni-WO_4_ than Ni-CO_3_ for all the 5000 cycles indicates that the pre-catalysts are not the true active sites but determine the final UOR activity.

## 3. Materials and Methods

### 3.1. Fabrication of β-Ni(OH)_2_, Ni-CO_3_, Ni-SiO_3_, Ni-MoO_4_, and Ni-WO_4_

1 M Ni^2+^, OH^−^, CO_3_^2−^, SiO_3_^2−^, MoO_4_^2−^, and WO_4_^2−^ solutions were prepared by dispersing the salts of NiCl_2_·6H_2_O, NaOH, Na_2_CO_3_, Na_2_SiO_3_, Na_2_MoO_4_·2H_2_O, and Na_2_WO_4_·2H_2_O in deionized water, respectively. *β*-Ni(OH)_2_ was obtained by mixing Ni^2+^ solution with OH^−^ solution with a volume ratio of 1:2. Ni-CO_3_, Ni-SiO_3_, Ni-MoO_4_, and Ni-WO_4_ were prepared by mixing Ni^2+^ solution with CO_3_^2−^, SiO_3_^2−^, MoO_4_^2−^, and WO_4_^2−^ solutions with a volume ratio of 1:1, respectively. After being thoroughly washed, centrifugated, and lyophilized, *β*-Ni(OH)_2_, Ni-CO_3_, Ni-SiO_3_, Ni-MoO_4_, and Ni-WO_4_ were obtained.

### 3.2. Structure Characterization

An X-ray diffractometer (XRD, DX–2700BH, Dandong Haoyuan instrument, Dandong, China) was used to collect XRD data with Cu K_α_ radiation at a scan rate of 2°·min^−1^. A transmission electron microscope (TEM, JEOL JEM–2100F, Japan Electronics Co., Ltd, Showima City, Tokyo, Japan) was used to observe catalysts’ morphologies with an accelerating voltage of 200 kV. An X-ray photoelectron spectroscope (XPS, Axis Supra, Shimadzu, Japan) was used to analyze element chemical states. A Shirley background was applied to all the XPS spectra and binding energies of all XPS spectra were charge referenced to 284.8 eV. The equation ∫*N*(*ε*)*ε*d*ε*/∫*N*(*ε*)d*ε* was applied to calculate the d band center, where *N(ε)* represents the density of states.

### 3.3. Electrochemcial Analysis

The UOR performance was evaluated in 1 M KOH with 0.33 M urea. The as-synthesized catalysts, a Hg/HgO electrode and a carbon rod, were used as the working, reference, and counter electrodes. Working electrodes were prepared by casting 30 μL catalyst inks onto a glass carbon electrode polished by a polishing cloth using 50 nm Al_2_O_3_. Catalysts (4 mg) and ketjen black (0.5 mg) dispersed into the mixed solution of ethanol (1000 μL) and Nafion (40 μL) were used as the catalyst ink. Cyclic voltammetry (CV) curves scanned at 5 mV·s^–1^, chronopotentiometry (CP) curves measured at 10 mA·cm^–1^, electrochemical impedance spectroscopy (EIS) tested at 1.4 V from 100 mHz to 100 kHz, and the rotating ring-disk electrode (RRDE) method were used to analyze the UOR performance. The electrochemical data were collected after 10 cycles of CV curves in 1 M KOH. All the potentials were calibrated by the equation *E_RHE_* = *E_Hg/HgO_* + 0.098 + 0.059 × pH. The reduction equivalent was calculated by the equation *RE* = n(Ni^3+^)/n(Ni^2+^).

## 4. Conclusions

In summary, *β*-Ni(OH)_2_, Ni-CO_3_, Ni-SiO_3_, Ni-MoO_4_, and Ni-WO_4_ were fabricated by bonding Ni^2+^ with OH^−^, CO_3_^2−^, SiO_3_^2−^, MoO_4_^2−^, and WO_4_^2−^ anions, respectively. Through systematic investigations about the catalysts structure and UOR mechanism, the five as-fabricated catalysts are found to serve as pre-catalysts and the true active sites are the in situ-formed Ni^3+^ moieties. The pre-catalyst has effects on the quantity of active sites and the activity per active site to determine the UOR activity. The as-fabricated amorphous nickel tungstate possesses the most superior activity per active site and a relatively high number of active sites, thus demonstrating excellent UOR activity to catalyze the UOR reaching 158.10 mA·cm^–2^ at 1.6 V, exceeding the majority of the reported catalysts. This work provides a deeper understanding of the pre-catalyst effect on UOR activity.

## Data Availability

The original contributions presented in the study are included in the article/Supplementary Material, further inquiries can be directed to the corresponding authors.

## Supplementary Materials

The following supporting information can be downloaded at: https://www.mdpi.com/article/10.3390/molecules29143321/s1, Figure S1: XRD patterns of β-Ni(OH)_2_, Ni-CO_3_, Ni-SiO_3_, Ni-MoO_4_ and Ni-WO_4_; Figure S2: (a–d) TEM images of *β*-Ni(OH)_2_, Ni-CO_3_, Ni-SiO_3_ and Ni-MoO_4_. (e–h) HRTEM images of *β*-Ni(OH)_2_, Ni-CO_3_, Ni-SiO_3_ and Ni-MoO_4_, insets are the corresponding SAED patterns; Figure S3: (a) Si 2p XPS of Ni-SiO_3_. (b) Mo 3d XPS of Ni-MoO_4_; Figure S4: (a) OER and UOR CV curves of *β*-Ni(OH)_2_, Ni-CO_3_, Ni-SiO_3_, Ni-MoO_4_, Ni-WO_4_ and 20 wt.% Ir/C. (b) OER CV curves of *β*-Ni(OH)_2_, Ni-CO_3_, Ni-SiO_3_, Ni-MoO_4_, Ni-WO_4_ and 20 wt.% Ir/C; Table S1: C 1s and W 4f XPS fitting results of Ni-CO_3_ and Ni-WO_4_.
