# Supplementary material for: Insights into the Understanding of the Nickel-Based Pre-Catalyst Effect on Urea Oxidation Reaction Activity
Source: Molecules. 2024 Jul 15;29(14):3321. doi: 10.3390/molecules29143321 (PMC11279396; doi:10.3390/molecules29143321)
Supplement: Supplementary file 1 [file molecules-29-03321-s001.zip › molecules-3096684-supplementary.pdf]

Supplementary Material:

Insights into the understanding of the nickel-based pre-catalyst effect on urea oxidation reaction activity

Haipeng Liu [1,2], Peike Wang [1,2], Xue Qi [1,2], Ao Yin [1,2], Yuxin Wang [1,2], Yang Ye [1,2], Jingjing Luo [1,2], Zhongqi Ren [1,2], Lina Chen [1,2,*], Suzhu Yu [1,2,*] and Jun Wei [1,2,3,*]

[1] Shenzhen Key Laboratory of Flexible Printed Electronics Technology, Harbin Institute of Technology (Shenzhen), Shenzhen 518055, China

[2] School of Materials Science and Engineering, Harbin Institute of Technology (Shenzhen), Shenzhen 518055, China

[2] State Key Laboratory of Advanced Welding and Joining, Harbin Institute of Technology (Shenzhen), Shenzhen 518055, China

* Correspondence: linachen@hit.edu.cn, szyu@hit.edu.cn and junwei@hit.edu.cn

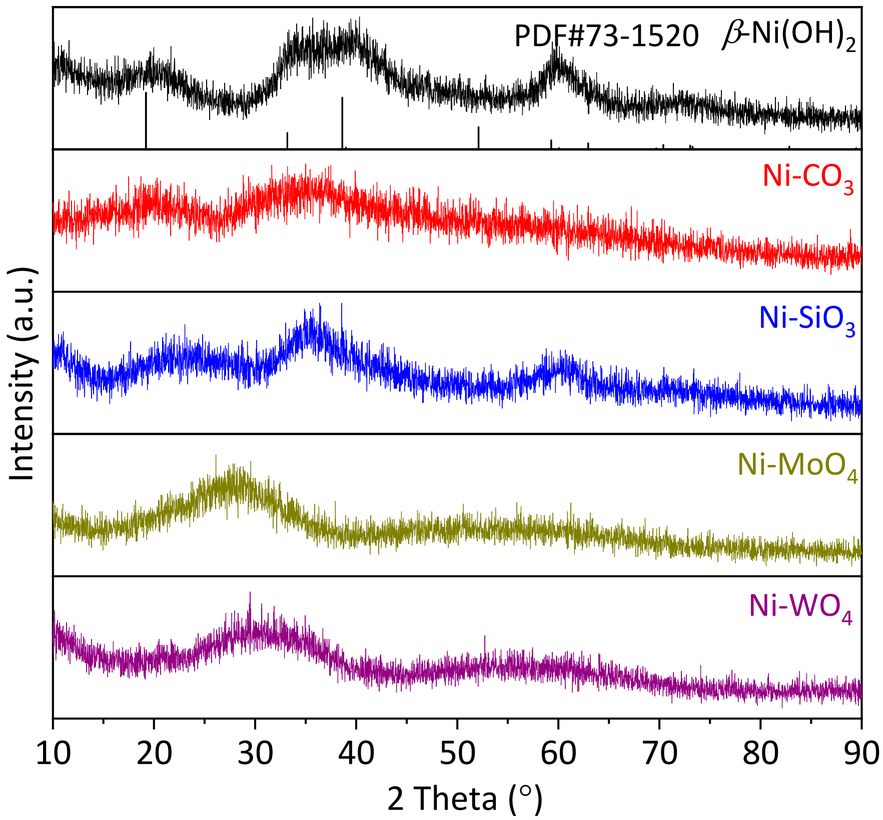

Figure S1 XRD patterns of *β*-$Ni(OH)_2$, Ni-CO_3, Ni-SiO_3, Ni-MoO_4 and Ni-WO_4.

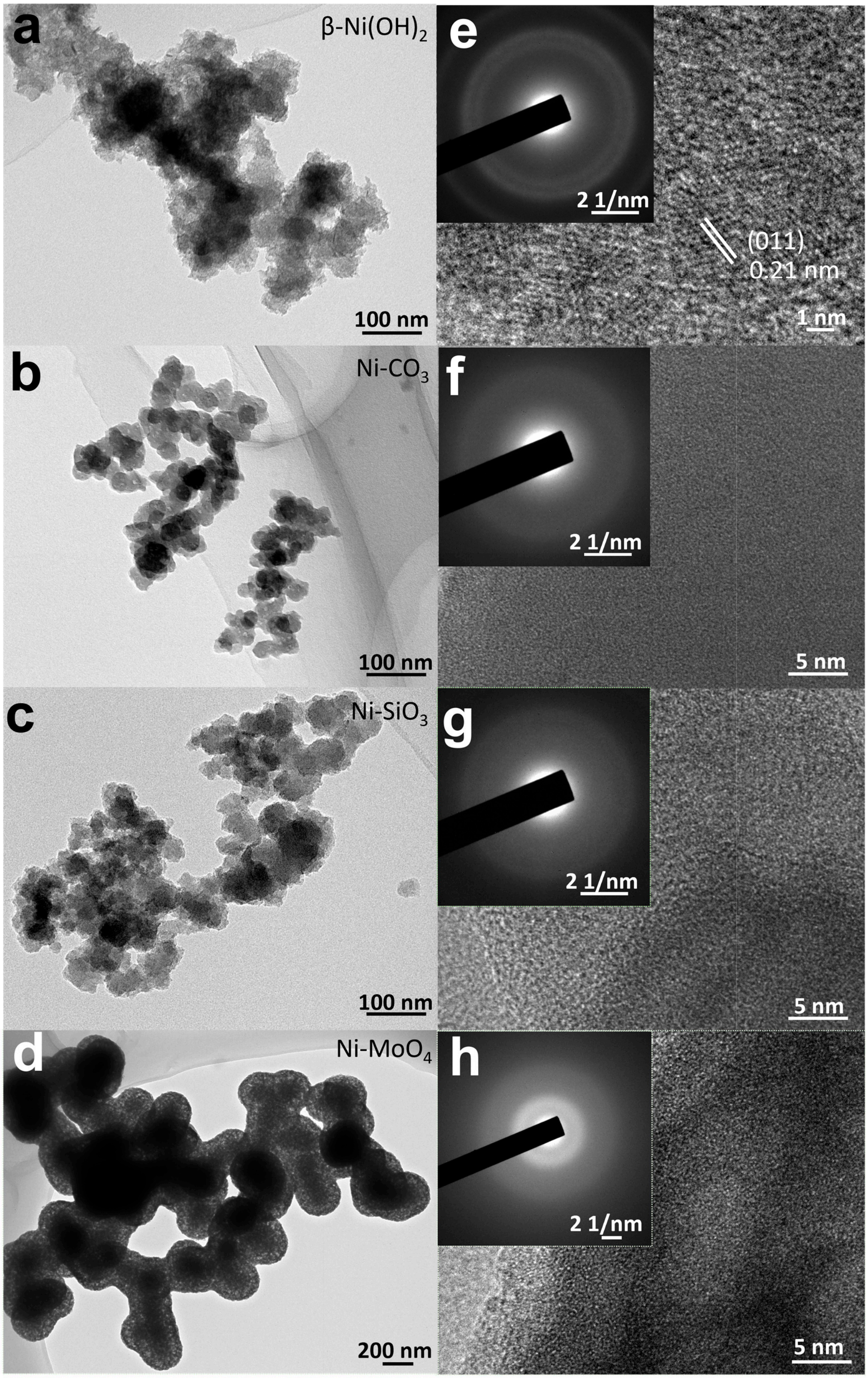

Figure S2 (a-d) TEM images of *β*-Ni(OH)$_2$, Ni-CO$_3$, Ni-SiO$_3$ and Ni-MoO$_4$. **(e-h)** HRTEM images of *β*-Ni(OH)$_2$, Ni-CO$_3$, Ni-SiO$_3$ and Ni-MoO$_4$, insets are the corresponding SAED patterns.

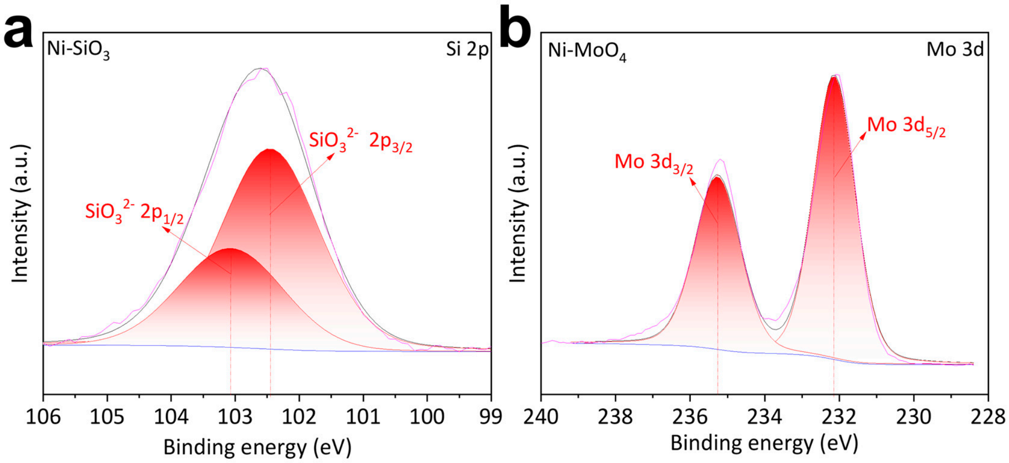

Figure S3 (a) Si 2p XPS of Ni-SiO_3. **(b)** Mo 3d XPS of Ni-MoO_4.

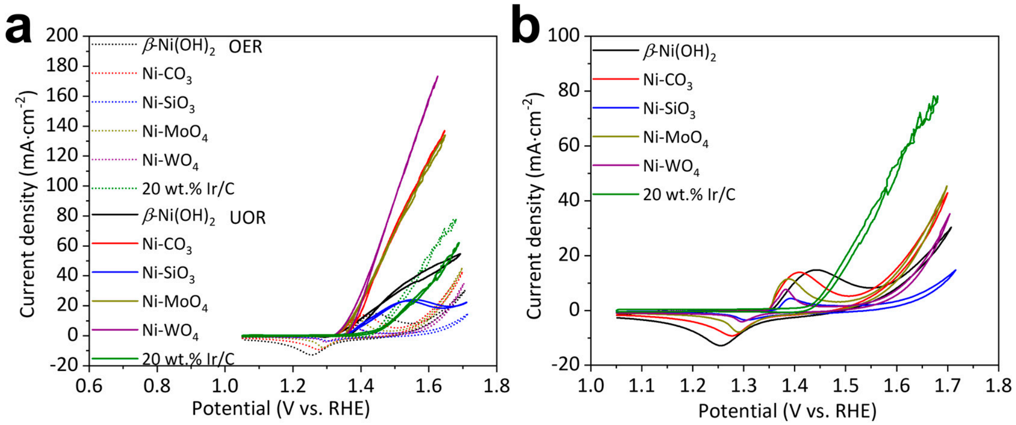

Figure S4 (a) OER and UOR CV curves of *β*-Ni(OH)$_2$, Ni-CO$_3$, Ni-SiO$_3$, Ni-MoO$_4$, Ni-WO$_4$ and 20 wt.% Ir/C. **(b)** OER CV curves of *β*-Ni(OH)$_2$, Ni-CO$_3$, Ni-SiO$_3$, Ni-MoO$_4$, Ni-WO$_4$ and 20 wt.% Ir/C.

Table S1 C 1s and W 4f XPS fitting results of $Ni\text{-}CO_3$ and $Ni\text{-}WO_4$

		$Ni\text{-}CO_3$	$Ni\text{-}CO_3$-durability	$Ni\text{-}WO_4$	$Ni\text{-}WO_4$-durability
W4f	WO^{4-} $4f_{7/2}$	–	–	34.98	–
	WO^{4-} $4f_{5/2}$	–	–	37.16	–
	Loss feature	–	–	40.81	–
C1s	Carbon C-C	284.60	284.60	–	–
	Carbon C-O	268.14	268.14	–	–
	Carbon C=O	287.60	287.60	–	–
	Carbon C-F	–	288.77	–	–
	CO_3^{2-} C-O	289.29	–	–	–
	CO_3^{2-} C=O	291.46	–	–	–
	$-CF_2-CF_2-/-SCF_2-C-$	–	292.19	–	–
	$-OCF_2-C-$	–	293.88	–	–
	$-CF_3-C-$	–	296.89	–	–
